# Reducing ghosting due to k-space discontinuities in fast spin echo (FSE) imaging by a new combination of k-space ordering and parallel imaging

**DOI:** 10.1016/j.jmr.2009.06.013

**Published:** 2009-09

**Authors:** David W. Carmichael, David L. Thomas, Roger J. Ordidge

**Affiliations:** a Department of Clinical and Experimental Epilepsy, UCL Institute of Neurology, London, UK; b Wellcome Trust Advanced MRI Group, Department of Medical Physics and Bioengineering, University College London, London, UK

**Keywords:** Magnetic resonance imaging, Fast spin echo, Turbo spin echo, High field, Artefacts, Parallel imaging, Point spread function, Ghosting

## Abstract

In multi-echo imaging sequences like fast spin echo (FSE), the point spread function (PSF) in the phase encoding direction contains significant secondary peaks (sidebands). This is due to discontinuities in adjacent k-space data obtained at different echo times caused by *T*_2_ decay, and leads to ghosting and hence reduced image quality. Recently, utilising multiple coils for signal reception has become the standard configuration for MR systems due to the additional flexibility that parallel imaging (PI) methods can provide. PI methods generally obtain more data than is required to reconstruct an image. Here, this redundancy in information is exploited to reduce discontinuity-related ghosting in FSE imaging. Adjacent phase encoded k-space lines are acquired at different echo times alternately in the regions of discontinuity (called ‘feathering’). This moves the resulting ghost artefacts to the edges of the field of view. This property of the ghost then makes them amenable to removal using PI methods. With ‘feathered’ array coil data it is possible to reconstruct data over the region of the discontinuity from both echo times. By combining this data, a significant reduction in ghosting can be achieved. We show this approach to be effective through simulated and acquired MRI data.

## 1 Introduction

To enable the acquisition of multiple phase encoding lines for each excitation of the magnetisation, many fast imaging methods utilise a train of echoes with data acquired at different echo times. One commonly used example is the fast spin echo sequence (FSE), where following spin excitation a train of RF refocusing pulses generates a set of spin echoes which are each differently phase encoded [1]. Typically, the signal amplitude of each echo in the train is predominantly modulated by *T*_2_ relaxation. This means that the k-space data has an effective filter applied across it along the phase encoding (PE) axis, which is mainly defined by *T*_2_ and the PE k-space ordering scheme. The resolution of each image pixel is defined by the point spread function (PSF), which is the Fourier transform (FT) of the effective k-space filter. Therefore, in FSE imaging, the PSF is degraded in the PE direction, resulting in artefacts and a loss of spatial resolution [2–4].

There are two main features of the PSF; (1) Broadening of the main peak in the PSF due to reduction of the echo amplitude via magnetisation decay along the echo train, and (2) The appearance of PSF sidebands due to discontinuities in k-space along the PE direction, which result from adjacent k-space lines obtained at different echo times being assigned to different portions of k_PE_-space. The result of this is often seen by a ‘ghosting artefact’ from regions of high signal intensity in the image repeated at intervals across the FoV. Parallel imaging has been used previously to reduce the full width at half maximum of the main peak [5]. The focus of this work is to minimise the k-space discontinuity-related ghosting (i.e. feature 2) for any given echo train.

In previous work [6], a method for dealing with the discontinuity-related ghosting called ‘feathering’ was introduced that utilised alternate lines of data (in the phase encoding direction) obtained at different echo times. This method reduced the overlap of the discontinuity-related ghosting with the image by altering the position of the largest ghost producing artefacts that were most significant at the edges of the field of view (FoV), necessitating over-sampling in the phase encoding direction with an associated cost in imaging time (although not in overall imaging efficiency).

More recently, the standard configuration for MR scanners has been modified to use a volume coil for RF transmission and a multiple receiver ‘array coil’ [7] for signal reception. This maximises the signal to noise ratio (SNR) for structural imaging. Parallel imaging (PI), which utilises array coils, has become an established method for speeding up image acquisition [8–11]. Related reconstruction methods have also been used for shortening EPI readout lengths [12] and for artefact reduction [13–17]. In this work, PI reconstruction methods, which are not normally useful in this context due to their SNR penalty, are integrated with ‘feathered’ k-space ordering to minimise ghosting artefacts caused by k-space discontinuities in FSE images. While this method has been used to reduce ghosting in our particular SNR optimised sequence, this method could be applied to any echo train.

## 2 Theory

**Fig. 1:**
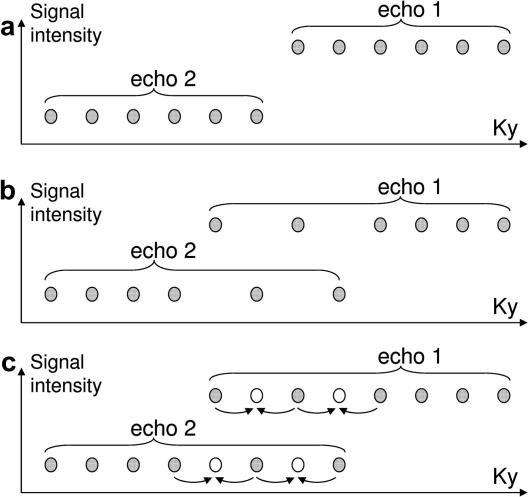
A representation of different PE ordering schemes with data from two echo times (a) A standard ordering scheme. (b) Feathered ordering scheme and (c) PI is used to reconstruct the open circles, obtaining two data points for the same position in k-space produced at different echo times. These are used to form two datasets, one containing the open circles and the second containing the filled circles in the ‘feathered’ region.

**Fig. 2:**
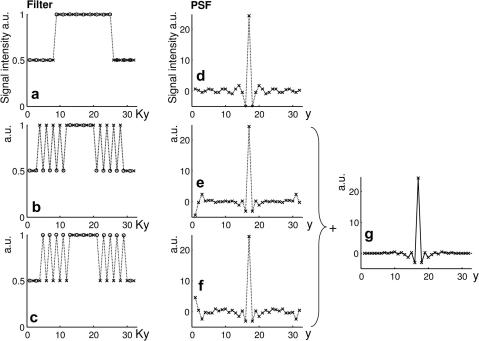
A simulation of the PSF from different PE ordering schemes. Data from two different echo times has different signal intensity leading to an effective filter along Ky (a–c). The FT of these filters gives the PSF (d–f). When the two offset feathered ordering schemes are averaged (g) an improved PSF is obtained without introducing side lobes at the edge of the field of view (along *y*). (a) A standard PE ordering scheme. (b) A feathered ordering scheme. (c) A feathered ordering scheme offset by one point along Ky. (d–f) The PSF obtained from the adjacent PE ordering scheme. (g) The average PSF of the two offset feathered PE ordering schemes (e–f).

In data acquired using the standard multi-shot FSE acquisition method, a discontinuity is present in the PE direction of k-space (Figs. 1a and 2a) due to lines of data being acquired at different echo times. This causes significant sidebands in the PSF (Fig. 2d). An overlapping region of k-space with data acquired alternately at different echo times (called ‘feathering’) can be used (Figs. 1b and 2b) [6]. This moves the sidebands in the PSF with strong intensity to the edge of the FoV (Fig. 2e). The ‘feathering’ corresponds to sub-sampling of the data at a particular echo time in the region of the k-space signal discontinuity. If a PI method such as GRAPPA [18] is used with the feathered data it is possible to obtain data from both echo times over the region of the discontinuity (Fig. 1c). Consequently two datasets can be produced, both of which contain the original data except in the region of the discontinuity (c.f. open and filled circles in Fig. 1c). These two complementary datasets can be combined to improve the PSF (and so reduce discontinuity-related ghosting in the image). One dataset uses the original feathering scheme (Fig. 2b with resulting PSF in Fig. 2e), whereas the other uses the GRAPPA-reconstructed feathering scheme that is offset in k-space by one point in the PE direction (Fig. 2c with resulting PSF in Fig. 2f). The PSF from each dataset is complementary, with the main sidebands adding destructively and the main peak adding constructively (Fig. 2g). This means that by combining the images obtained from each dataset, the main discontinuity-related ghost can be removed. It is noted that the addition of the two images with different PSFs is the same as adding the two complex k-space datasets together before performing the FT. The resulting k-space filter would contain an intermediate signal level within the feathered region as if the data in the feathered region came from an intermediate echo time.

An alternatively way of combining the two data sets is to perform a weighted average in the region of the k-space discontinuity. The result of this is similar to echo time shifting [19] because the steps in the k-space filter are replaced with a gradual transition. However, in this case it is performed in post processing from a single feathered data set from multiple coils instead of altering pulse sequence timing.

## 3 Materials and methods

### 3.1 Simulation details

All code was written in Matlab (www.mathworks.com). A simulation was written where a 1D PE k-space filter was simulated as a matrix of 32 points. To simulate data from two echo times, half of these points were given a signal level of one, while the rest were given a signal level of 0.5. The k-space order of the data was altered (as in Fig. 1) to create one ‘standard’ filter applied along the PE direction of k-space (Ky) and two ‘feathered’ filters, one offset by one point along Ky with respect to the other (see Fig. 2b and c). The PSF was calculated by taking the FT of the filter.

A detailed simulation was performed of the k_PE_-space filter applied in our FSE sequence, optimised for brain imaging at 4.7 T [6,20], and the effect of this filter on image artefact (see Fig. 2). The sequence (modelling the experiment described below) used an echo train length of eight echoes with 22 ms separation and with coherence of spin echo and stimulated echo pathways maintained to reduce signal decay during the echo train. The refocusing RF pulses used were 163° creating a stimulated echo component of magnetisation along the *z*-axis that contributes to subsequent echoes in the train. This was simulated using the extended phase graph algorithm description of spin magnetisation in the presence of multiple RF pulses [21–23]. Full longitudinal relaxation of magnetisation was assumed during the TR; brain *T*_2_ values were estimated as 60 ms from the literature [24,25]. The signal decay at the eight different echo times was calculated using an arbitrary signal level of 100 immediately after excitation of the magnetisation by a simulated 90° RF pulse. To create the k-space filters the calculated signal decay during the echo train was combined with different PE ordering schemes. Three schemes were used; (a) a standard centric PE scheme, (b) the feathered scheme used in experiments below, (c) the feathered scheme offset by one PE line.

The ‘mean artefact power’ was calculated as the sum of the magnitude of the PSF outside the main peak on one side of the (symmetric) function. Finally, the ‘maximum artefact power’ was obtained by measuring the maximum peak height of the PSF outside of the central peak wherever it occurred.

### 3.2 Experimental details

Experiments were performed on a SMIS MR5000, 4.7 T/90 cm bore system supported by Philips Medical Systems, utilising a head gradient set (Magnex, Oxford, UK) and a standard birdcage coil for RF transmission. Healthy subjects were imaged with informed consent and local ethics approval. A custom 4-channel head array coil (PulseTeq Ltd., www.pulseteq.com) was used for RF reception [20]. The FSE sequence has been previously described [6]. Here, a resolution of 352 × 352 × 2000 μm was used. To obtain this resolution in the readout direction the receiver bandwidth was increased while the readout duration was kept the same (i.e. avoiding increased inter-echo spacing). Due to the limited receiver bandwidth options available on our system, the bandwidth had to be doubled (from 50 to 100 kHz). In the phase encoding direction ‘feathering’ was used [6]. This method normally requires over-sampling in the phase encode direction; a factor of 3/2 was used here. With over-sampling of 3/2 times in both directions the FoV was 360 × 270 mm. Images were not cropped, leaving the over-sampled regions to view any artefacts present there. Removing the requirement for this PE over-sampling (and thereby minimising the total scan time) is one of the main goals of this work. Other parameters were TR = 3.5 s, matrix = 1024 × 768, and 17 axial slices with an interslice gap of 2 mm; total acquisition time was 5 min 40 s. The sequence employed a pulse train of eight 163° refocusing pulses following the initial 90° excitation pulse, forming eight echoes spaced 22 ms apart with effective TE = 22 ms. Phase consistency was maintained between echoes within the echo train by phase correction based on a non-phase encoded reference scan and a Hanning filter was applied prior to image reconstruction (FT).

**Fig. 3:**
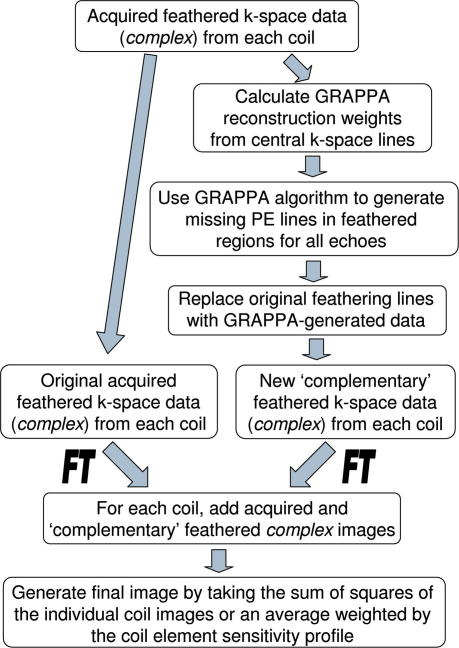
A flow chart demonstrating the required data processing steps.

The ‘missing data’ in the feathered region was reconstructed using a two block, two column GRAPPA [18] algorithm, thereby obtaining data from each echo time over the ‘feathered’ region. This data was combined in two different ways. Firstly, two feathered datasets offset by one point along Ky were obtained by taking different points (like the open and filled circles in Fig. 1c) in the feathered region. These images were FT’d and then the complex images summed. The square root of the sum of squares (SSQ) of the individual coil images was taken as shown in Fig. 3.

## 4 Results

### 4.1 Simulation

The results of the simulation are shown in Fig. 2; on the left hand side is the simulated k-space filter and on the right hand side is the real part of the FT of the filter. In Fig. 2a, the effective PE filter of the standard FSE acquisition is demonstrated, with the corresponding PSF in Fig. 2d. Due to the discontinuity in the filter, there are large oscillations in the PSF stretching across the FoV. Feathering was applied, using alternate echo times for adjacent Ky points (Fig. 2b and c). The feathering alters the side lobes in the PSF, greatly reducing the size of the side lobes next to the main peak (Fig. 2e and f), but producing significant lobes at the edges of the FoV. The two different feathered filters (offset by one position along Ky) produce PSF functions that are very similar. However, the strong lobes at the edges of the FoV have opposite sign. When these two PSF functions were averaged, the main peak added constructively but the outer lobes cancelled (Fig. 2g). This improved PSF would be produced by averaging two images with offset feathering.

### 4.2 Simulation of the 4.7 T FSE sequence

**Fig. 4:**
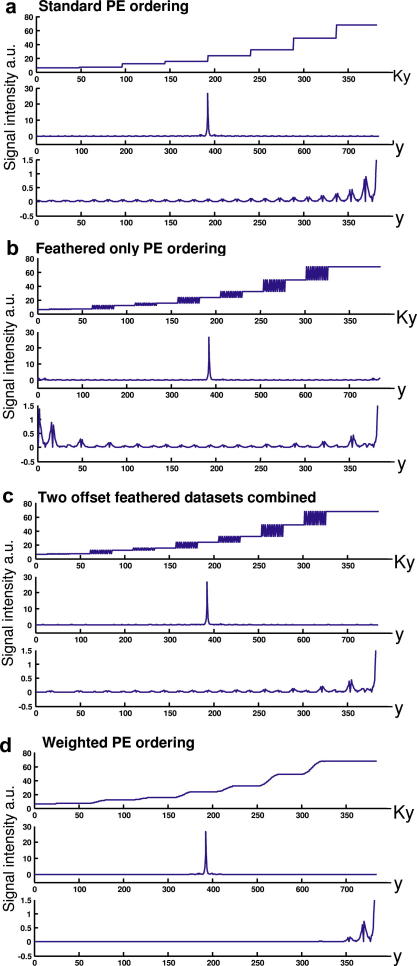
A simulation of the effective k-space filter and resulting PSF from different PE ordering schemes and data reconstruction methods for the FSE sequence optimised for neuroimaging at 4.7 T. (a) A standard PE ordering scheme is shown for half the phase encoding steps (1–384 of 768) at the top with successive steps in the filter from different echo times. In the middle the magnitude of the PSF is displayed. At the bottom, half of the PSF magnitude plot is displayed with a different scale to show the side lobes. (b) As ‘a’ except a feathered PE ordering scheme is used. The resulting PSF has smaller side lobes, however, large intensity remains at the edge (e.g. *y* = 0–20). (c) As ‘b’ except two datasets are produced containing feathering offset by one point along Ky (top). This produces a PSF with lower side lobes without producing large intensities at the edge. (d) As ‘c’ except using a weighted combination of the two datasets to change the effective filter (top). The PSF from this filter is also greatly improved; the number and size of the side lobes is reduced. However, the first side lobe is larger than for the combined feathered data in ‘c’.

The results of the simulation of the PSF for the FSE pulse sequence used on our 4.7 T system are shown in Fig. 4. In each of the Fig. 4a–d, the effective k-space filter from the edge of k-space to the centre (i.e. Ky 1–384 of 768 PE lines) is shown at the top of three plots. In the middle, the full PSF is displayed, and at the bottom, half of the PSF is shown with the signal intensity rescaled to view the side lobes. In Fig. 4a, the PSF without feathering shows a degraded PSF with large side lobes over a large spatial extent. In Fig. 4b, feathering is simulated with the oscillations present in the k-space filter. The resulting PSF is altered, with reduced side lobes. However, large lobes remain at the edge of the FoV (low *y*). By combining two datasets offset by one point along Ky (Fig. 4c), the improved performance of the feathered PSF is maintained close to the central peak, while the large lobes shifted to the edge of the FoV are removed. Finally, in Fig. 4d the filter is altered by performing a weighted average of the two datasets, resulting in smooth transitions between data obtained at different echo times. The resulting PSF is improved with slightly smaller side lobes adjacent to the main peak of the PSF and the spatial extent of the PSF greatly reduced. In Table 1, the mean artefact power is lowest from performing a weighted average. However, this power is concentrated over a smaller spatial extent with the peak artefact power twice as high as for the combined feathered dataset. The application of a Hanning filter increases the mean artefact power, particularly for the weighted filter while the peak artefact power remains similar. The combined feathered PSF should produce the least visible artefacts due to the lower peak artefact power. For comparison, the primary peak intensity (see Fig. 4) is 25.3 a.u. hence the largest ghost would be reduced from 3.6% of the intensity of the primary image for a standard scheme to 1.7% for the combined feathered dataset.

### 4.3 Imaging results

**Fig. 5:**
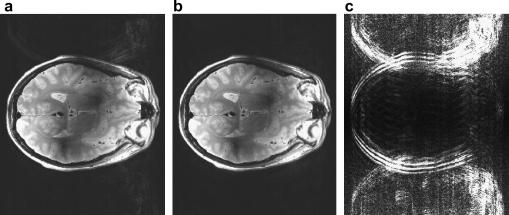
Artefact removal in feathered FSE images. Two images were reconstructed from a single feathered dataset by using GRAPPA to create a second image with feathering offset by one point along Ky. By adding these two images together the artefact is removed due to the improvement in the PSF by subtracting the images the removed artefact can be seen. (a) SSQ image obtained using the feathering method. (b) The magnitude image of the sum of the complex images with offset feathering. (c) The magnitude of the difference between the original image (a) and the combined image (b). N.B. The same data is used to reconstruct images ‘a and b’.

**Fig. 6:**
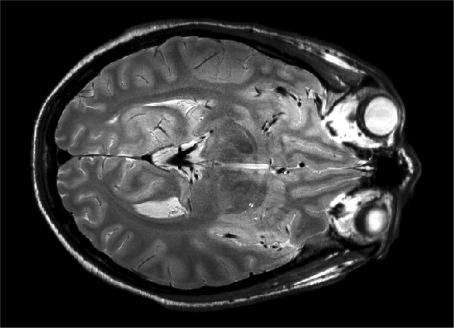
An intensity corrected FSE image using the offset feathered data created from a single dataset using GRAPPA. The image is the same as Fig. 5e. However, the individual coil images have been optimally weighted and summed according to the sensitivity of the individual coils (i.e. SENSE processing with no speed up). The image demonstrates high quality and a low artefact level.

From one full feathered dataset, two datasets were produced using GRAPPA, with feathering in each dataset offset by a one point along Ky. The images produced are displayed in Fig. 5. Ghost artefacts from k-space discontinuities were visible in the magnitude images from the individual feathered datasets in both the single coil images and the square root of the sum of squares (SSQ) image (Fig. 5a). By summing the complex images from the offset feathered datasets these ghost artefacts were removed (Fig. 5b). This is due to the effect on the PSF seen in Fig. 4. To clearly view the difference between the original SSQ image and the new image they were subtracted and displayed (Fig. 5c). The ‘ghosting’ seen in this difference image, which is primarily from the shifted large sidebands in the PSF of the feathering method, has been removed. The artefact is predominantly outside the object, due to the large field of view used in the PE direction. If a standard FoV (e.g. 220 mm) were used in the PE direction, with feathering alone, significant artefact would lie within the object. Finally, a rescaled intensity corrected, cropped image is shown in Fig. 6 (using entirely the same data as in Fig. 5a). The image appears of high quality with little observable artefact remaining.

## 5 Discussion

At higher field strength (⩾3T) the PSF in the PE direction of the FSE pulse sequence is degraded because *T*_2_ becomes shorter. Methods typically used to reduce this problem are reducing the inter-echo spacing and increasing the number of refocusing pulses to prevent large discontinuities [2–5]. However, the resulting increase in SAR is often unacceptable at higher field strength where operation at the regulatory limits is often necessary for useful FSE sequences. One option would be to reduce the echo spacing and the refocusing flip angle. However, reducing the refocusing flip angle would increase the size of the first step in the effective filter mitigating any reduction in the resulting ghost intensity. Although reducing the echo spacing is an effective method for improving the PSF (FWHM), discontinuities which produce ghosting are relatively unaffected because the decay is exponential and reduced echo spacing requires that sampling is performed during the steepest part of the decay. Hence even where reduced echo spacing is employed, discontinuity-related ghosting artefacts may still be a significant problem and can be reduced with the method proposed here. Smoother signal weighting can be obtained by averaging different k-space ordering schemes [26]. However, the cost of performing this operation is dependant on the time at which the centre of k-space must be sampled for optimal CNR. We have previously shown for the human brain at 4.7 T [6] that the best CNR was obtained at a short echo time (22 ms) using the first echo in the echo train. This means that if averaging was performed with opposite phase encode ordering a considerable cost in SNR per unit time (of nearly 50%) would be incurred. An additional penalty in CNR would result because the effective echo time would be non-optimal. Hence averaging additional scans with different k-space ordering schemes are of limited benefit in this context. Echo time shifting [19] can also used; however, this necessitates a longer echo train duration costing valuable imaging time and is not compatible with a ‘balanced’ FSE sequence (using stimulated echo pathways in addition to the spin echo to compensate for *B*_1_ non-uniformity). One method for reducing this ghosting is to use feathering, where adjacent k-space lines are obtained with alternating echo times. This shifts the majority of the ghosting by half the FoV. This necessitates over-sampling in the phase encoding direction to avoid ghost artefacts overlying the image [6]. While improving image SNR, this PE over-sampling significantly increases imaging time.

Here, feathering was combined with PI methods so that ghost artefacts from k-space discontinuities could be reduced with no cost in imaging time. This was achieved using GRAPPA to reconstruct two datasets from a single acquired dataset containing feathering. In each of these, the feathered region has been offset by one point along Ky. The resulting ghost from each dataset is complementary, being of identical magnitude and opposite phase. This means that, when the complex images produced from each of the two datasets are summed, the ghost artefacts cancel leaving an image with reduced artefact. This removes the previous requirement for over-sampling when the feathering method was used. There is little cost in SNR from this process because only a few lines of k-space are reconstructed with GRAPPA in the regions where the feathering is applied (away from the centre of k-space). Ample calibration data is automatically obtained from the fully sampled k-space centre.

The combination of GRAPPA and feathering generates data from two echo times over the feathered region. An alternative approach for using this data is to perform a weighted average of the two datasets in k-space, creating a smooth transition between data from different echo times. This mimics echo time shifting [19], with the advantages that the phase coherence of both spin echo and stimulated echo pathways can more easily be maintained to compensate *B*_1_ inhomogeneity and no increase in echo train duration is required. Both the combined feathering and the weighted average datasets have significantly reduced ghosting for our implementation of the FSE at 4.7 T (see Figs. 4, 5 and Table 1). The ‘combined feathered’ datasets produced the smallest amplitude side lobes while the weighted average produced the lowest overall ‘artefact power’. Both approaches will improve the resolution of the FSE particularly at higher field strengths. The images produced with the array coil have a resolution of 352 × 352 × 2000 μm and an average signal to noise ratio (SNR) of approximately 20. This is a higher resolution than previously published by our group [6,20]. At this resolution and SNR, small anatomical details of the brain are visible such as cortical myeloarchitecture (see Fig. 6). The improvement in image resolution obtained using the methodology presented here is incremental, but significant in scale when compared to fine structural details present in these images. A larger reduction in ghosting may be possible if larger regions of k-space were feathered. However, an improvement in the PSF will have an associated cost in SNR and artefact level because more lines will be reconstructed with GRAPPA.

Previous work to remove artefacts with PI were mainly focused on EPI Nyquist ghost artefacts (and required an *a priori* model of a ‘pseudo PSF’ that contained the Nyquist ghost) [13] and motion artefacts [14–17]. In this work, ghost artefacts are removed without a model of the PSF; instead the PSF is manipulated by using both PI reconstruction methods and PE k-space ordering. The idea of integrating k-space sampling and PI has previously been applied to alter the aliasing patterns produced by undersampling, and to improve image reconstruction by reducing the local enhancement of noise (i.e. improving the g-factor) [27]. PI methods allow an ‘extra degree of freedom’ when optimising imaging strategies. Here, ghosting due to k-space discontinuities in a multi-echo sequence is controlled, allowing a greater choice of parameters such as inter-echo spacing and number of refocusing pulses. These factors (in part) determine SAR, resolution, SNR and image contrast, so greater flexibility in their choice is advantageous.

To remove the sharp steps in the effective k-space filter, echo time shifting [19] can be used. However, echo time shifting with an FSE sequence that maintains coherence of spin echo and stimulated echo pathways to reduce signal decay during the echo train produces coherences with a temporal offset during the readout which produce image artefacts. Additionally, echo time shifting only reduces discontinuity-related ghosting to a moderate degree and involves an overhead in both imaging time and sequence complexity. One further approach is to use variable flip angle RF pulses and use ‘transition between pseudo steady states’ (TRAPS) [28] or ‘hyperechoes’ [29] to create long echo trains with a relatively smooth transition between echo times and a large reduction in SAR. These sequences are predominantly used to reduce RF power deposition (SAR), allowing longer echo trains to be utilised. There is some penalty in SNR, particularly for the echoes used to encode the outer PE lines in the echo train, which when combined with relatively fast changes in signal intensity with PE k-space position, can lead to some loss in resolution. Additionally, these methods entail an increase in sequence complexity in terms of pulse sequence design [28,29] and contrast behaviour [30]. Finally, because these methods rely on a transition between pseudo steady states, very large spatial variations in *B*_1_ found at higher fields (⩾4.7 T) mean that the pseudo steady state cannot always be achieved across the imaging volume of interest.

This method may be applied to reduce discontinuity-related ghosting in other multi-echo sequences. In particular, 3D experiments will benefit, where both large signal discontinuities occur along the second PE axis causing large PSF degradation and ‘balanced’ sequences are desirable to minimise signal decay during long echo trains. The method presented here can be used in conjunction with other methods such as TRAPS [28] and hyperechoes [29] to reduce discontinuity-related ghosting in FSE imaging while limiting SAR.

### 5.1 SNR

The reduction in SNR by using GRAPPA will depend on coil/slice/PE geometry and on the exact GRAPPA reconstruction used. However, the SNR penalty from reconstructing a small number of off-centre k-space lines space with an effective speed up factor of two (i.e. alternate k-space lines) is small. If more central k-space lines are reconstructed using GRAPPA (e.g. if a shorter echo train were to be used with a reduced matrix size) a larger reduction in SNR can result. There are a large number of factors to be considered when choosing an optimised echo train for maximum contrast and signal to noise ration (SNR). The approach we have taken is to maximise SNR, with the motivation of imaging at the highest resolution possible in a feasible scan time for patients (∼12 min for whole brain coverage). To maximise SNR we have chosen: (1) High field strength to maximise SNR, (2) Relatively low receiver bandwidth (100 kHz), (3) A ‘balanced’ sequence maximal magnetisation from both stimulated and spin echoes are refocused, (4) maximal flip angle for refocusing pulses (163°). By using a low receiver bandwidth and large refocusing flip angles, a relatively long inter-echo spacing is required. The consequence of this maximum SNR approach is that the k-space discontinuities are large while the overall decay is minimised (by having fewer steps in total). Finally it must be remembered that intolerance to spatial variations in *B*_1_ is very important for high field pulse sequences, hence the approach described here represents an alternative high SNR, *B*_1_-insensitive method [6] at higher field strengths.

## 6 Conclusions

In multi-echo imaging sequences like FSE, the point spread function in the phase encoding direction is significantly degraded. This is due to discontinuities in adjacent k-space data obtained at different echo times caused by *T*_2_ decay which result in ghost artefacts. To moderate these effects, phase encoding lines of data (in k-space) were acquired at different echo times alternately (called ‘feathering’) in the regions of discontinuity. When used with PI methods, data are reconstructed over the region of the discontinuity from both echo times. These data were combined in two different ways; (a) by performing an FT on two feathered datasets offset by one point along Ky before adding the complex images, and b) by performing a weighted average of the two datasets (in k-space) to create a smooth transition in signal. Both of these methods significantly reduced discontinuity-related ghosting with no cost in imaging time (i.e. neither over-sampling nor echo time shifting is needed and no prior information is used). This method provides a greater flexibility in the design and optimisation of the FSE sequence and may be used to reduce k-space discontinuity-related ghosting in other multi-echo sequences.

## Figures and Tables

**Table 1:** Comparison of the artefact from different PE ordering/reconstruction schemes.

	All a.u.		Hanning filter applied	
	Peak after power	Mean artefact power	Peak after power	Mean artefact power
Standard	0.9	19.46	0.89	19.84
Combined feathered	0.43	10.91	0.43	10.98
Weighted average	0.74	5.42	0.72	8.12
